# Tooth-Ache as a Cause of Paralysis

**Published:** 1880-02

**Authors:** T. H. Parramore

**Affiliations:** Hampton, Va.


### ARTICLE VII.

### *Tooth-ache as a Cause of Paralysis.*

BY T. H. PARRAMORE, D. D. S., HAMPTON, VA.

That tooth-ache is often caused by morbid conditions of other organs, and *vice versa*, no one who has studied this subject can deny.

Dr. C. A. Harris, in writing of the effect of tooth-ache upon remote parts of the body, says: "To quote the language of Mr. Bell, 'I have seen this occur not only in the face, over the scalp, in the ear, and underneath the lower jaw; but down the neck, over the shoulder, and along the whole length of the arm.'" No writer, however, that I remember having read, mentions paralysis as one of the effects of tooth-ache, and speaks of its cure being effected by the removal of the offending member. I met with a case of this kind a short time ago, and thinking it might be of interest to your readers, determined to report it.

Mrs. M., aged 30, of sanguine temperament and good health, sent for me to extract her teeth. Upon looking into her mouth I found she had lost several teeth, and her mouth was in such a condition as to necessitate the removal of all her upper and several of her lower teeth. My question elicited the following information with regard to her case: whenever she had tooth-ache she suffered terribly from what she called "cramp" of the right arm and side, stating that her arm was frequently drawn up behind her back further than she could possibly reach by her utmost exertion. This condition would continue for several hours, when this side of her body (from her neck nearly to her hips) would become paralysed, and had at one time continued paralysed for six weeks; but had never failed to be relieved by the extraction of the aching tooth. She has never borne children, but has had several abortions. Her husband is still living. I found the extraction of each of her teeth followed by rigidity of the muscles of her right arm, (described by her as "cramp;") this soon passed off and was followed by a pricking sensation, which she described by saying her arm and hand were "asleep," showing a decided tendency to paralysis of the brachial plexus. In conversation with a physician of over forty years experience in active practice, I mentioned this case, when he informed me that he had met with three similar cases and each had resisted all his efforts to effect a cure until he extracted all decayed teeth and roots, when, in each instance, the paralysis was relieved in a very short time. The pathology of this case puzzles me. I would like to hear from men eminent in our profession upon it. Can we settle it by saying it is reflex action? Can reflex action account for all the conditions? Or could irritation of the trifacial (arising as it does, by two roots, which have been traced into the spinal cord) so effect that organ as to produce paralysis of the cervical and brachial plexuses? These are questions entirely too deep for me. But I am convinced that it is only by close study of such cases as the one described above that we can ever hope to arrive at a correct understanding of the functions of the different parts of the brain and the intricate relations existing between the different nerves, both cranial and spinal.

